# Association between *Helicobacter pylori* Infection and Risk of Osteoporosis in Elderly Taiwanese Women with Upper Gastrointestinal Diseases: A Retrospective Patient Record Review

**DOI:** 10.1155/2014/814756

**Published:** 2014-05-13

**Authors:** Shih-Chun Lin, Malcolm Koo, Kun-Wei Tsai

**Affiliations:** ^1^Department of Geriatrics, Dalin Tzu Chi Hospital, Buddhist Tzu Chi Medical Foundation, 2 Minsheng Road, Dalin, Chiayi 62247, Taiwan; ^2^Department of Medical Research, Dalin Tzu Chi Hospital, Buddhist Tzu Chi Medical Foundation, 2 Minsheng Road, Dalin, Chiayi 62247, Taiwan; ^3^Dalla Lana School of Public Health, University of Toronto, 155 College Street, 6th Floor, Toronto, ON, Canada M5T 3M7

## Abstract

*Introduction. Helicobacter pylori (H. pylori)* infection could lead to chronic local and systemic immune response. The resulting increase in proinflammatory cytokines could affect bone resorption and might increase the risk of osteoporosis. This study aimed to investigate the association between *H. pylori* infection and osteoporosis in elderly female patients with upper gastrointestinal diseases. *Methods*. A retrospective patient record review study was conducted in a regional teaching hospital in south Taiwan. Relevant information on female patients aged 65 and over who were diagnosed with diseases of esophagus, gastric ulcer, or duodenal ulcer during January 2008 to December 2010 were abstracted. Association between *H. pylori* infection and osteoporosis was evaluated using multivariate logistic regression analysis. *Results*. Of the 365 patients with a mean age of 77.3 years, 77 (21.1%) had *H. pylori* infection and 101 (27.7%) had been diagnosed with osteoporosis. Multivariate logistic regression analysis revealed that osteoporosis was significantly associated with *H. pylori* infection (adjusted odds ratio = 2.03, 95% confidence interval = 1.14–3.62) after adjusting for age group, body mass index group, and use of proton pump inhibitor. *Conclusion*. Osteoporosis was found to be associated with *H. pylori* infection in Taiwanese female patients with upper gastrointestinal diseases. Further studies with information on potential confounders are needed to confirm the association.

## 1. Background

Osteoporosis is a major public health problem affecting up to one in three women [1]. It has been estimated that approximately half of the hip fractures worldwide are a result of osteoporosis [2]. The morbidity, disability, and mortality associated with osteoporosis can impose a substantial burden on affected individuals, their families, and the health care system [3]. Therefore, identifying risk factors and at risk populations for osteoporosis are important for the prevention of hip fracture.

*Helicobacter pylori* (*H. pylori*) is a spiral-shaped, Gram-negative bacterium that colonizes the gastric mucosa of more than 50% of human population. It can elicit lifelong inflammatory and immune responses in infected individuals. It is strongly associated with chronic gastritis, peptic ulcer, gastric mucosa-associated lymphoid tissue (MALT) lymphoma, and gastric cancer [4]. In addition,* H. pylori* infection has previously been suggested to be involved in the pathogenesis of a number of extradigestive disorders including cardiovascular, skin, rheumatic, liver cirrhosis, diabetes mellitus, and respiratory diseases [5].

*H. pylori* infection can increase the systemic levels of inflammatory cytokines and bone turnover regulating cytokines such as tumor necrosis factor-alpha, interleukin-1, and interleukin-6 [6]. Since these factors are involved in the pathogenesis of osteoporosis, it is plausible that individuals with* H. pylori* infection are at an increased risk of osteoporosis. However, few studies have investigated this association except in a study of 80 osteoporotic male patients and 160 controls where the results showed that individuals infected by CagA-positive* H. pylori* strains was more prevalent in men with osteoporosis [7]. On the other hand,* H. pylori* infection was not accompanied by significant changes in biochemical markers of bone metabolism in a study of 41* H. pylori* positive children [8]. In addition, to our knowledge, only one study has specifically evaluated the association in elderly females [9]. Therefore, using retrospective patient medical record analysis, we investigated the association between* H. pylori* infection and osteoporosis among elderly Taiwanese women with upper gastrointestinal diseases.

## 2. Methods

### 2.1. Study Design and Data Collection

A retrospective patient medical record analysis was performed using the data on the computerized patients' record database in a regional hospital in south Taiwan. The study protocol was approved by the institutional review board of the Dalin Tzu Chi Hospital, Buddhist Tzu Chi Medical Foundation (no. B10003001). Informed consent was not required because of the nature of the study. Female patients were included in the study if they were 65 years or older and were diagnosed with diseases of esophagus, gastric ulcer, or duodenal ulcer (International Classification of Diseases, Ninth Revision, Clinical Modification [ICD-9-CM] codes of 530.xx to 532.xx) between January 1, 2008, and December 31, 2010.

A medical record abstraction form was used to collect information on age, body weight, height, smoking, alcohol use,* H. pylori* testing, diagnosis of osteoporosis, the use of proton pump inhibitor (PPI), and antiosteoporotic medications. The presence of* H. pylori* infection was determined by histological examination [10], rapid urease test [11], or both. Body mass index (BMI) was calculated as the weight in kilograms divided by the height in meters squared. It was further divided into four categories according to the classification of the Department of Health of Taiwan: underweight (BMI < 18.5 kg/m^2^), normal weight (BMI = 18.5–23.9 kg/m^2^), overweight (BMI = 24.0–26.9 kg/m^2^), and obese (BMI ≥ 27 kg/m^2^). Osteoporosis was defined by either dual energy X-ray absorptiometry (DXA) scan [12] or use of osteoporosis medications. The latter was mainly as a result of bony fractures related to osteoporosis rather than use for primary prevention of osteoporosis.

### 2.2. Statistical Analyses

Categorical variables were expressed as frequencies and percentages and Fisher's exact test was used for their comparisons. Multiple logistic regression analyses were used to evaluate the association between osteoporosis and* H. pylori* infection, adjusting for age, BMI, and the use of proton pump inhibitor. Hosmer-Lemeshow statistics were used to assess model fit. A two-tailed *P* value <0.05 was considered statistically significant.

## 3. Results

A total of 671 female patients with upper gastrointestinal diseases were identified from the medical records. Of them, 494 (73.6%) had undergone testing for* H. pylori* and a further 129 patients were excluded from the final analysis due to missing data in other biochemical variables. The mean age of these 365 patients was 77.3 years (median = 77, range = 65–97 years). Of them, 77 (21.1%) were tested positive for* H. pylori* and 101 (27.7%) had been diagnosed with osteoporosis.

Table 1 compares the characteristics between patients tested positive and negative for* H. pylori*. The group tested negative for* H. pylori* was younger (*P* = 0.061) and had a higher proportion with gastric ulcer (*P* = 0.051). The group tested positive for* H. pylori* had significantly higher proportion of duodenal ulcer (*P* = 0.033). No significant differences were observed in the distributions of tertiles of PPI use between the two groups. Results from multivariate logistic regression analyses revealed that osteoporosis was significantly associated with* H. pylori* infection (adjusted odds ratio = 2.03, 95% confidence interval = 1.14–3.62) after adjusting for age group, body mass index group, and use of proton pump inhibitor (Table 2).

## 4. Discussion

The key finding of retrospective patient medical record analysis was that* H. pylori* infection was significantly associated with osteoporosis in elderly Taiwanese women with upper gastrointestinal diseases. The odds of osteoporosis in patients tested positive for* H. pylori* infection were twice of that tested negative and the association was independent of age, BMI, and PPI use.

To date, there was only one cross-sectional study designed to specifically evaluate the association between* H. pylori* infection and osteoporosis in postmenopausal women [9]. The authors of the study concluded that* H. pylori* infection was not significantly different between patients with or without osteoporosis (*P* = 0.22). Nevertheless, with only 50 subjects, the study suffered from a low statistical power to detect the association when it truly exists (power = 33%). Studies with adequate sample sizes are needed to ensure that a reasonable statistical power can be achieved.

Although the exact mechanisms underlying the observed association between* H. pylori* infection and osteoporosis are not clear, several possible explanations have previously been postulated. First, chronic infection of* H. pylori* can increase the release of cytokines, such as tumor necrosis factor- (TNT-) *α*, interleukin-1, and interleukin-6. It has been demonstrated that increased levels of interleukin-1 and TNF-*α* can lead to bone resorption [13] and, therefore, may increase the risk of osteoporosis.

In addition,* H. pylori* infection has shown to decrease the levels of vitamin B_12_ [8, 14]. Low levels of vitamin B_12_ are considered as a risk factor for osteoporosis [15, 16]. Furthermore, medications for the treatment of upper gastrointestinal disorders such as PPI might affect calcium absorption and thereby increase the risk of osteoporosis. A prospective cohort study conducted over a period of 10 years in Canada reported that PPI use was associated with significantly lower baseline body mineral density at the femoral neck and total hip. However, PPI use over 10 years did not appear to be associated with accelerated loss of bone mineral density [17]. A review study on 14 observational studies published from 1980 to early 2011 concluded that PPI use was associated with a modest increase in the risk of hip fracture and vertebral fracture. Nevertheless, some studies showed conflicting results, possibly as a result of residual confounding [18]. In our study of patients with upper gastrointestinal diseases, PPI use was not significantly associated with osteoporosis. A possible reason is that long-term use of PPI is not under the coverage of the Taiwan National Health Insurance scheme and, therefore, patients generally use it for only 2 to 4 months. PPI use was also not significantly different between* H. pylori* positives and negatives for the same reason of lack of long-term insurance coverage.

Findings from this study should be weighed within the confines of the limitations of our data source. First, the study subjects came from a single regional hospital, which might not be representative of other settings. Second, retrospective studies based on abstraction of medical records are constrained by the accuracy and completeness of the records. Third, not all patients with upper gastrointestinal diseases were tested for presence of* H. pylori*. Therefore, we could not rule out the presence of selection bias. Fourth, measurement of systemic inflammatory markers was not available to confirm whether they were elevated in patients with* H. pylori* infection.

## 5. Conclusions

Results from our retrospective patient medical record analysis indicated that* H. pylori* infection was associated with osteoporosis in elderly female Taiwanese. Further studies using prospective design with information on systemic inflammatory markers and potential confounding factors are needed to confirm the association.

## Figures and Tables

**Table 1:** Characteristics of patients according to *H. pylori* infection.

Variable	Frequency (%)			*P*
	Total	*H. pylori* positive	*H. pylori* negative	
	365 (100)	77 (21.1^a^)	288 (78.9)	
Age (years)				0.061
65–74	145 (39.7^b^)	22 (28.6)	123 (42.7)	
75–84	150 (41.1)	36 (46.8)	114 (39.6)	
≥85	70 (19.2)	19 (24.7)	51 (17.7)	
Body mass index^c^				0.938
Underweight	39 (11.7)	9 (13.2)	30 (11.4)	
Normal	153 (46.1)	30 (44.1)	123 (44.1)	
Overweight	73 (22.0)	16 (23.5)	57 (23.5)	
Obese	67 (20.2)	13 (19.1)	54 (19.1)	
Smoking				0.742
Yes	13 (3.6)	3 (3.9)	10 (3.5)	
No	351 (96.4)	74 (96.1)	277 (96.5)	
Alcohol use				>0.999
Yes	6 (1.6)	1 (1.3)	5 (1.7)	
No	358 (98.4)	76 (98.7)	282 (98.3)	
Diseases of esophagus (ICD-9-CM^d^ code 530)				0.695
Yes	144 (39.5)	32 (41.6)	112 (38.9)	
No	221 (60.5)	45 (58.4)	176 (61.1)	
Gastric ulcer (ICD-9-CM code 531)				0.051
Yes	216 (59.2)	38 (49.4)	178 (61.8)	
No	149 (40.8)	39 (50.6)	110 (38.2)	
Duodenal ulcer (ICD-9-CM code 532)				0.033
Yes	56 (15.3)	18 (23.4)	38 (13.2)	
No	309 (84.7)	59 (76.6)	250 (86.8)	
Proton pump inhibitor use				0.289
Not use	23 (6.3)	2 (2.6)	21 (7.3)	
<2 month	115 (31.5)	23 (29.9)	92 (31.9)	
≥2 months	227 (62.2)	52 (67.5)	175 (60.8)	

^a^Row percentage.

^b^Column percentage.

^c^33 missing values in body mass index, 1 missing value in smoking and alcohol use.

^d^International Classification of Diseases (ICD), Ninth Revision (ICD-9), Clinical Modification.

**Table 2:** Multivariate logistic regression analysis of osteoporosis (*N* = 332).

Variable	*n* (%)	Odds ratio (95% CI)	*P*
Age (years)			
65–74	138 (41.6)	1.00	
75–84	132 (39.8)	1.06 (0.62–1.82)	0.838
≥85	62 (18.7)	0.93 (0.45–1.90)	0.830
Body mass index group			
Normal	153 (46.1)	1.00	
Underweight	39 (11.8)	0.62 (0.27–1.43)	0.264
Overweight	73 (22.0)	0.49 (0.24–0.96)	0.039
Obese	67 (20.2)	0.95 (0.51–1.79)	0.881
Proton pump inhibitor use			
Not use	23 (6.9)	1.00	
<2 month	101 (30.4)	1.33 (0.44–4.02)	0.610
≥2 months	208 (62.7)	1.32 (0.46–3.79)	0.606
*H. pylori *			
Negative	264 (79.5)	1.00	
Positive	68 (20.5)	2.03 (1.14–3.62)	0.016

Since there were no drinkers or smokers in the patients with osteoporosis in our study, their associations with osteoporosis were not analyzed.

Only 332 subjects were included in the final model due to 33 missing values in body mass index.

## Abbreviations

BMI: Body mass index
*H. pylori*: * Helicobacter pylori*
PPI: Proton pump inhibitor.
